# Remote EMDR versus CBT for PTSD after the Kahramanmaraş earthquakes: a randomized trial

**DOI:** 10.3389/fpsyt.2026.1779057

**Published:** 2026-05-22

**Authors:** Metin Çınaroğlu, Eda Yılmazer, Selami Varol Ülker, Gökben Hızlı Sayar

**Affiliations:** 1 Psychology Department, Faculty of Administrative and Social Science, İstanbul Nişantaşı University, İstanbul, Türkiye; 2 Psychology Department, Faculty of Social Science, Beykoz University, İstanbul, Türkiye; 3 Faculty of Humanities and Social Sciences, Üsküdar University, İstanbul, Türkiye; 4 Medical School, Head of Institute of Social Science, Üsküdar University, İstanbul, Türkiye

**Keywords:** cognitive behavioral therapy, earthquake, EMDR, post-traumatic stress disorder, randomized controlled trial

## Abstract

**Background:**

Large-scale earthquakes are associated with high and persistent rates of post-traumatic stress disorder (PTSD), depression, and anxiety. Although Eye Movement Desensitization and Reprocessing (EMDR) and Cognitive Behavioral Therapy (CBT) are both guideline-recommended treatments for PTSD, direct randomized comparisons—particularly in post-disaster settings and via remote delivery—remain limited.

**Methods:**

In this randomized, controlled, parallel-group trial (ClinicalTrials.gov Identifier: NCT06758362), 89 adult survivors of the 2023 Kahramanmaraş earthquakes in Türkiye who met DSM-5 criteria for PTSD were assigned to 12 sessions of remotely delivered EMDR (n = 30), CBT (n = 30), or a wait-list control group (n = 29). PTSD severity was the primary outcome, with depression, anxiety, and emotion regulation as secondary outcomes. Assessments were conducted at baseline, mid-treatment, and post-treatment. Analyses followed an intention-to-treat framework using mixed-effects models.

**Results:**

Both EMDR and CBT produced large reductions in PTSD symptoms compared with the control condition (Cohen’s d ≈ 1.9), alongside moderate-to-large improvements in depression (d ≈ 1.4) and anxiety (d ≈ 1.3). Symptom improvement was evident by mid-treatment and increased through post-treatment. Direct comparisons between EMDR and CBT yielded overlapping confidence intervals, indicating comparable overall efficacy. However, EMDR showed a tendency toward greater reduction in core PTSD symptoms, whereas CBT demonstrated relatively stronger effects on depressive symptoms. Improvements in emotion regulation were observed in both treatment groups but did not reach statistical significance.

**Discussion:**

The findings suggest that both EMDR and CBT are highly effective when delivered remotely in the aftermath of a large-scale natural disaster. Differential response patterns likely reflect distinct therapeutic mechanisms—direct trauma memory processing in EMDR versus cognitive restructuring and behavioral activation in CBT—highlighting their potential complementarity rather than superiority of one approach over the other. The absence of spontaneous improvement in the control group underscores the necessity of structured psychological intervention following earthquake exposure.

**Conclusion:**

Remotely delivered EMDR and CBT are feasible, effective, and clinically valuable interventions for earthquake-related PTSD. Their comparable efficacy and partially distinct symptom profiles support flexible, needs-based integration of both treatments within disaster mental-health systems.

**Clinical Trial Registration:**

https://clinicaltrials.gov/study/NCT06758362, identifier NCT06758362.

## Introduction

Natural disasters—particularly earthquakes—are sudden, unpredictable events that profoundly disrupt both the physical environment and the psychological and social fabric of communities (1, 2). Their lack of warning and immediate threat to survival make earthquakes especially potent triggers for trauma-related psychopathology (3). Survivors are frequently exposed to multiple stressors, including bereavement, injury, displacement, destruction of homes, and prolonged uncertainty (4), with post-traumatic stress disorder (PTSD) consistently emerging as one of the most prevalent and debilitating outcomes (5). Global studies estimate that 10–40% of earthquake survivors develop PTSD, often accompanied by depression, anxiety, and emotional dysregulation, leading to long-term functional impairment and increased healthcare use (61, 6–8). Recent studies following major earthquakes have similarly documented high rates of PTSD, depression, anxiety, sleep disturbance, and broader psychosocial impairment among survivors and healthcare workers (56–63). In Türkiye, a region of high seismic activity, the 2023 Kahramanmaraş earthquakes caused unprecedented destruction and psychological trauma across 11 provinces (9, 10). Over 50,000 deaths, millions displaced, and extensive social disruption (11, 12) have underscored the urgent need for scalable, evidence-based psychological interventions tailored to earthquake survivors’ complex and ongoing stressors. Research examining the structural, social, and healthcare consequences of the 2023 Kahramanmaraş earthquakes has further emphasized the scale and complexity of post-disaster recovery needs in Türkiye (64–69).

### Psychological sequelae of earthquake exposure

Post-traumatic stress disorder (PTSD) is among the most persistent and disabling psychological outcomes following major natural disasters such as earthquakes. It involves intrusive memories, nightmares, physiological hyperarousal, and pervasive threat perception that disrupt emotional, social, and occupational functioning (13–15). Survivors may experience emotional numbing, loss of trust, and maladaptive beliefs about safety and control (16). In earthquake contexts, PTSD is intensified by the cumulative and prolonged nature of exposure. Survivors face both the acute terror of ground shaking and the chronic stress of displacement, bereavement, resource scarcity, and recurring aftershocks (17, 18). The persistence of instability and shared loss fosters ongoing hypervigilance, helplessness, and emotional dysregulation, often leading to secondary symptoms such as depression, anxiety, and insomnia (19, 20). These patterns underscore the urgent need for interventions that address the complex, enduring, and context-specific nature of post-earthquake PTSD.

### Evidence-based psychotherapeutic interventions

In response to the growing mental health burden following disasters, several psychological interventions have been empirically supported for post-traumatic stress disorder (PTSD) (21). Among them, trauma-focused cognitive behavioral therapy (CBT) and Eye Movement Desensitization and Reprocessing (EMDR) are the most widely endorsed and guideline-recommended treatments (22). Both target unprocessed traumatic memories and maladaptive coping but differ in theoretical focus and technique. CBT is grounded in cognitive-behavioral models emphasizing the restructuring of trauma-related beliefs, reduction of avoidance, and acquisition of adaptive coping skills. It combines psychoeducation, graded exposure, and cognitive restructuring to restore emotional regulation and a sense of control (23). EMDR, by contrast, facilitates adaptive reprocessing of traumatic memories through bilateral stimulation—such as eye movements or tactile tapping—without requiring detailed verbal recounting. This approach can be particularly effective for individuals who struggle with emotional articulation or dissociation. Both therapies show strong efficacy for PTSD, yet few studies have directly compared their effectiveness in large-scale disaster contexts where psychological distress is pervasive (24).

### Research gap and rationale

Although both CBT and EMDR are well supported for trauma-related disorders, direct comparisons in large-scale, real-world disasters remain limited. Most prior studies have examined a single modality, used small or non-randomized samples, or lacked control groups, and the majority were conducted in high-income clinical settings, limiting generalizability to resource-constrained or culturally distinct contexts such as post-earthquake Türkiye. Moreover, few investigations have compared these therapies across broader symptom domains—such as comorbid depression, anxiety, and emotion regulation—among survivors of complex, cumulative trauma (25). Evidence for remote delivery of these interventions is also scarce (26, 27), despite its growing necessity in disaster settings where infrastructure damage, displacement, and therapist shortages restrict access to care. The 2023 Kahramanmaraş earthquakes created precisely such conditions, underscoring the need to evaluate the clinical effectiveness of online EMDR and CBT to inform scalable, evidence-based psychological responses in future disaster contexts.

### Study aim and hypotheses

The present study aimed to evaluate and compare the effectiveness of Eye Movement Desensitization and Reprocessing (EMDR) and Cognitive Behavioral Therapy (CBT) in reducing symptoms of post-traumatic stress disorder (PTSD), depression, anxiety, and emotion regulation difficulties among adult survivors of the 2023 Kahramanmaraş earthquakes in Türkiye. Utilizing a randomized controlled trial design with a no-treatment control group, the study assessed outcomes at three critical time points: before the intervention (T1), after the sixth therapy session (T2), and following the twelfth and final session (T3). All interventions were delivered remotely via secure online platform to ensure accessibility and continuity of care in the disaster-affected regions. It was hypothesized that both EMDR and CBT would result in significant reductions in PTSD, depression, and anxiety symptoms compared to the control group. Additionally, it was expected that EMDR would yield stronger improvements in core PTSD symptoms due to its focus on direct memory processing, while CBT would lead to greater improvements in depressive and anxiety symptoms, as well as emotion regulation, given its structured emphasis on cognitive restructuring and behavioral activation. This study represents one of the first randomized controlled comparisons of EMDR and CBT in a post-earthquake population within a remote therapy format, aiming to provide critical insight for scalable, evidence-based psychological interventions in disaster settings.

## Methods

### Study design

This randomized, controlled, open-label, parallel-group clinical trial was conducted to evaluate the comparative effectiveness of EMDR and CBT for PTSD symptoms among adult survivors of the 2023 Kahramanmaraş earthquakes in Türkiye. Participants were randomly allocated using a computer-generated sequence by an independent researcher to one of three arms: EMDR (n = 30), CBT (n = 30), or a waitlist control group (n = 29). Due to the nature of psychotherapeutic interventions, no blinding of participants or therapists was implemented (open-label design); however, data analysis was conducted by a blinded assessor. Both interventions were delivered remotely via Microsoft Teams over 12 weekly sessions. Outcome measures were assessed at three time points: baseline (pre-treatment), midpoint (after the 6th session), and endpoint (after the 12th session). The timing of these assessments was standardized relative to both randomization and session count to ensure temporal comparability across groups. Randomization (T0) occurred within one week of the baseline assessment, and nearly all participants (94%) commenced therapy within 7–10 days thereafter. As each session was held weekly, midpoint assessments (T2) occurred approximately 6–7 weeks and post-treatment assessments (T3) approximately 12–13 weeks after randomization. The waitlist control group was assessed at equivalent intervals (T2 ≈ 7 weeks, T3 ≈ 13 weeks post-randomization) to maintain equal elapsed time across study arms. Analysis of mean days from randomization to each assessment confirmed no significant between-group differences at T2 or T3. A follow-up evaluation is planned and will be reported separately. This study adheres to the CONSORT guidelines for reporting randomized controlled trials, and a detailed CONSORT flow diagram outlining participant enrollment, allocation, retention, and analysis is provided in the **Supplementary 1**. Follow-up assessments were conducted online by an independent clinical psychology graduate assistant who was not involved in treatment delivery and remained blinded to participant group allocation throughout the study to minimize assessment bias. Randomization (T0) was conducted within one week following baseline assessment. Participants initiated treatment within approximately 7–10 days post-randomization. Mid-treatment assessments (T2) were conducted after the 6th session (approximately 6–7 weeks post-randomization), and post-treatment assessments (T3) after the 12th session (approximately 12–13 weeks post-randomization). The wait-list control group was assessed at equivalent time intervals to ensure temporal comparability across groups.

### Power analysis

*A priori* power analysis was conducted using G*Power 3.1 to determine the required sample size for detecting a medium effect size in a three-arm randomized controlled trial (EMDR, CBT, and control). Assuming a repeated-measures ANOVA framework with three time points (pre-treatment, mid-treatment, and post-treatment), an alpha level of.05, power (1–β) set at.80, and an expected medium effect size (f = 0.25; equivalent to η² = .06), the analysis indicated a total sample size of 78 participants (26 per group) would be sufficient to detect statistically significant between-group differences over time. To ensure sufficient power while accounting for potential attrition, recruitment was extended to reach 90 participants across the three groups (n = 30 EMDR, n = 30 CBT, n = 30 control). This exceeded the minimum threshold for statistical power, ensuring adequate sensitivity for detecting clinically meaningful differences between the intervention and control groups. The final sample size (N = 89) exceeded the minimum required sample (N = 78), ensuring adequate statistical power for detecting group × time interaction effects.

### Inclusion and exclusion criteria

Participants were eligible for inclusion if they (a) were between the ages of 18 and 65, (b) had directly experienced the 2023 Kahramanmaraş earthquakes, (c) met the DSM-5 criteria for Post-Traumatic Stress Disorder (PTSD) based on clinical interview and score thresholds on the PTSD Checklist for DSM-5 (PCL-5), and (d) had access to a stable internet connection to attend remote therapy sessions via Microsoft Teams. Exclusion criteria included (a) current diagnosis of a psychotic disorder, bipolar disorder, or severe cognitive impairment, (b) acute suicidality or substance dependence requiring immediate medical intervention, (c) ongoing psychiatric treatment or psychotherapy initiated within the past three months, and (d) significant hearing or visual impairments that would interfere with participation in video-based sessions. Individuals who were unable to provide informed consent or who were currently receiving disability benefits for psychiatric reasons were also excluded to minimize confounding clinical variables.

### Randomization and blinding

Participants were randomly allocated using a computer-generated sequence created by an independent researcher not involved in recruitment or treatment delivery. Allocation concealment was ensured by separating the randomization process from assessment and intervention procedures. Due to the nature of psychotherapeutic interventions, participants and therapists could not be blinded to treatment condition. Outcome data were collected via online self-report measures. An independent research assistant, who was not involved in treatment delivery and remained unaware of group allocation, coordinated the assessment process, including distributing assessment links and monitoring completion. Although no clinician-rated outcomes were used, this procedure ensured that assessment administration and data handling were conducted independently of treatment allocation. In addition, statistical analyses were conducted on datasets in which group labels were coded to minimize bias.

### Participants

Participants were recruited from regions directly affected by the 2023 Kahramanmaraş earthquakes through a diverse outreach strategy, including private outpatient psychiatric clinics, psychotherapy centers, earthquake-related psychological support hotlines, post-earthquake WhatsApp community groups, and personal networks of clinical psychology graduate students living in the region. Recruitment targeted the following cities: Kahramanmaraş, Hatay, Kilis, Malatya, Gaziantep, Diyarbakır, Elazığ, Adıyaman, Osmaniye, Adana, Şanlıurfa, Batman, Kayseri, Mardin, Bingöl, Tunceli, and Niğde. To ensure ecological validity, individuals who relocated to other cities after the earthquake were excluded. The initial target sample was 150 individuals exhibiting PTSD symptoms related to the earthquake who had not received psychotherapy or psychiatric medication. All interested individuals completed an online diagnostic interview using the *Structured Clinical Interview for DSM-5 PTSD* (SCID-5-CV), administered by the first three authors—assistant professors in clinical or applied psychology—under the supervision of the senior author, a professor of psychiatry. Participants who met inclusion criteria were randomly assigned to one of three groups: EMDR therapy, CBT therapy, or a wait-list control. Details of participant flow, attrition, and final sample composition are presented in the *Results* section and summarized in the CONSORT diagram (**Supplementary 1**). Participants who discontinued the study were retained in ITT analyses up to the point of withdrawal, with missing observations handled within the MMRM framework.”

### Intervention

The study included two active treatment arms—EMDR and CBT—and a no-treatment control group. Participants in the intervention arms received 12 individual weekly sessions, each approximately 50 minutes in duration, delivered remotely via Microsoft Teams. The decision to conduct therapy online was based on both practical and clinical considerations. The Kahramanmaraş earthquakes affected 11 cities in southeastern Türkiye, while the therapists were based in Istanbul. To ensure equitable access and continuity of care for all participants regardless of location, a standardized online format was adopted. This approach also minimized logistical barriers and adhered to national and international health guidelines for remote mental health services.

#### EMDR therapy group

the EMDR intervention followed the standardized eight-phase protocol developed by Shapiro. These phases included: (1) history taking and case conceptualization, (2) preparation and stabilization, (3) assessment of traumatic memories, (4) desensitization using bilateral stimulation (BLS), (5) installation of positive cognitions, (6) body scan for residual somatic distress, (7) closure, and (8) re-evaluation. Bilateral stimulation was adapted for the online format through screen-based visual tracking (e.g., therapist-guided finger or object movement) and client-administered tactile techniques such as alternating tapping (e.g., the butterfly hug method). The feasibility and effectiveness of EMDR delivered via online have been supported by recent empirical studies, particularly in trauma-exposed populations. Meta-analytic evidence suggests that online EMDR is associated with significant reductions in PTSD symptoms, comparable to in-person treatment (28). The EMDR International Association (EMDRIA) also endorses telehealth delivery under appropriate conditions. All EMDR sessions in this study were conducted in accordance with these established standards. The intervention followed the standard eight-phase model described by Shapiro (29) and aligned with the EMDR International Association (30). Treatment fidelity was monitored in accordance with EMDRIA competency criteria.

#### CBT therapy group

The CBT intervention followed a structured, trauma-focused cognitive behavioral therapy protocol delivered over 12 weekly individual sessions (approximately 50 minutes each). The protocol was manual-informed and based on established trauma-focused CBT frameworks (31, 32), adapted for remote delivery. The intervention was organized into three phases.

In the early phase (Sessions 1–4), the focus was on psychoeducation about trauma and its psychological effects, normalization of symptoms, and the development of coping skills. Participants were introduced to the cognitive model of PTSD, and initial techniques included relaxation training, grounding exercises, and basic behavioral activation to reduce avoidance and increase daily functioning. In the middle phase (Sessions 5–8), treatment emphasized cognitive restructuring and the identification of maladaptive trauma-related beliefs (e.g., guilt, helplessness, overgeneralized threat). Therapists guided participants in recognizing automatic thoughts, evaluating cognitive distortions, and generating alternative, more adaptive interpretations. Behavioral experiments and continued activation strategies were used to reinforce cognitive change. In the late phase (Sessions 9–12), exposure-based techniques were introduced, including imaginal exposure to trauma-related memories and gradual *in vivo* exposure to avoided situations, where clinically appropriate. These sessions also focused on emotional processing, relapse prevention, and consolidation of skills. Participants were encouraged to integrate cognitive and behavioral strategies into daily life and to develop individualized coping plans for future stressors.

Throughout the intervention, structured worksheets and homework assignments were used to reinforce session content and promote skill generalization. All materials were adapted for secure online delivery, and homework was reviewed at the beginning of each session. Treatment fidelity was monitored through regular supervision and standardized rating procedures.

#### Control group

The control group received no therapeutic intervention during the 12-week study period. Participants in this group underwent the same assessment schedule as the intervention groups and were offered psychological support or referral upon completion of the study, if requested. A wait-list control design was selected due to ethical considerations in a post-disaster context, ensuring that all participants could access psychological support after the study period.

To enhance reproducibility and comparability across treatment arms, both interventions followed structured, manual-informed protocols with predefined session objectives. Session progression was standardized across participants, while allowing limited flexibility to address individual clinical needs. All sessions were delivered via a secure online platform, and adherence to protocol was monitored throughout the study using standardized fidelity procedures. Participants in the control group were instructed not to initiate any new psychotherapy or structured psychological treatment during the 12-week study period and were informed that they would be offered psychological support following study completion. At each assessment point, participants were asked whether they had engaged in any external psychological or psychiatric treatment. No participants reported initiating formal psychotherapy during the study period.

### Therapists, supervision, and quality assurance

The psychotherapy sessions were delivered by trained clinicians with formal certification in their respective therapeutic modalities. All EMDR sessions were conducted by the second author, who holds an official EMDR therapy certification and has experience working with trauma-exposed populations. The CBT sessions were delivered by the first and third authors, both of whom have completed certified training programs in cognitive behavioral therapy and have prior clinical experience in CBT-based interventions. All treatment procedures were supervised by the senior author, a professor of psychiatry with over 20 years of clinical and academic experience. The supervisory process included regular case discussions, protocol adherence checks, and ethical oversight throughout the course of the study. To ensure treatment fidelity and methodological rigor, a quality assurance committee was formed. This committee consisted of one board-certified psychiatrist, one clinical psychologist with advanced expertise in CBT, and one clinical psychologist experienced in EMDR. As part of the fidelity monitoring process, three video recordings (each 10–15 minutes in duration) were randomly selected from each participant’s session series and reviewed by the committee. The committee used structured fidelity checklists derived from the EMDR Fidelity Rating Scale (33) and the Cognitive Therapy Rating Scale (34), both adapted for online delivery and standardized in Turkish. Each session segment was rated on a 0–2 scale for adherence to protocol, therapist competence, and session integrity (0 = not demonstrated, 1 = partial, 2 = full adherence). Across all rated segments, mean fidelity scores ranged between 1.8 and 2.0 (SD < 0.3) for both EMDR and CBT, indicating high procedural adherence and therapist competence. Detailed scores and reviewer comments are available in **Supplementary 2**. Feedback was provided to the study therapists when necessary to maintain consistency and protocol compliance across all participants. This multi-tiered supervision and fidelity monitoring framework ensured that the interventions were delivered with high methodological and clinical standards, supporting both internal validity and ethical integrity. Because therapists were nested within treatment conditions (i.e., EMDR and CBT delivered by different clinicians), therapist effects cannot be fully disentangled from treatment effects. However, standardized supervision, protocol adherence checks, and formal fidelity monitoring were implemented to minimize variability across interventions. Within the CBT condition, participants were distributed approximately equally between the two therapists to minimize therapist-specific bias.

### Measurements

Psychological assessments were conducted at three time points: baseline (prior to the intervention), mid-treatment (after the 6th session), and post-treatment (after the 12th session). The following standardized self-report instruments were administered online in validated Turkish versions to evaluate PTSD symptoms, depression, anxiety, and emotion regulation difficulties.

Primary and secondary outcomes: the primary outcome of the study was the severity of post-traumatic stress symptoms, as measured by the PCL-5. This measure directly reflects the principal therapeutic target of both EMDR and CBT interventions. The secondary outcomes included depression (BDI-II), anxiety (BAI), and emotion regulation difficulties (DERS), which were evaluated to capture broader changes in affective and regulatory domains associated with trauma recovery.

PTSD checklist for DSM-5 (PCL-5): the primary outcome measure was the PCL-5, a 20-item self-report scale developed by Blevins et al. (2015) to assess the presence and severity of PTSD symptoms according to DSM-5 criteria. The scale is structured around four symptom clusters: intrusion, avoidance, negative alterations in cognition and mood, and hyperarousal. Items are scored on a 5-point Likert scale from 0 (“Not at all”) to 4 (“Extremely”), yielding a total score range of 0 to 80. A cut-off score of 33 is generally accepted to indicate probable PTSD. The Turkish adaptation was performed by Boysan et al. (35), showing strong internal consistency and validity. Prior EMDR and CBT studies have reported large effect sizes (Cohen’s d = 1.2 to 2.0) for treatment-related reductions in PCL-5 scores.

Beck depression inventory-II (BDI-II): depressive symptoms were assessed using the BDI-II, developed by Beck, Steer, and Brown (1996). This 21-item inventory measures cognitive, affective, and somatic symptoms of depression over the previous two weeks. The instrument comprises two main factors: cognitive-affective and somatic symptoms. Each item is rated on a 4-point scale from 0 to 3, with total scores ranging from 0 to 63. Scores above 17 typically indicate at least mild depressive symptoms. The Turkish version was validated by Kapcı et al. (36), demonstrating high internal reliability. Treatment studies report moderate to large pre-post effect sizes (Cohen’s d = 0.8 to 1.5) for BDI-II scores.

Beck Anxiety Inventory (BAI): anxiety symptoms were evaluated with the BAI, developed by Beck, Epstein, Brown, and Steer (1988). The BAI includes 21 items, each scored on a 4-point scale (0 to 3), with a total score range of 0 to 63. The instrument primarily measures physiological and subjective symptoms of anxiety, and factor analyses often support either a unidimensional or two-factor structure (somatic vs. subjective components). A cut-off of 16 or higher is commonly used to indicate moderate anxiety. The Turkish adaptation was carried out by Ulusoy et al. (37). Treatment outcome studies show that BAI scores typically demonstrate significant improvement, with effect sizes ranging between d = 0.7 and 1.3.

Difficulties in Emotion Regulation Scale (DERS): emotion regulation difficulties were assessed using the DERS, developed by Gratz and Roemer (2004). The DERS consists of 36 items across six subscales: Nonacceptance of emotional responses, Difficulties engaging in goal-directed behavior, Impulse control difficulties, Lack of emotional awareness, Limited access to emotion regulation strategies, and Lack of emotional clarity. Each item is rated on a 5-point Likert scale, yielding total scores ranging from 36 to 180, with higher scores indicating greater emotion dysregulation. The Turkish adaptation was performed by Yiğit & Yiğit (38), and the scale has demonstrated good psychometric properties. While no universal clinical cut-off exists, higher scores are consistently linked with psychopathology. Intervention studies report effect sizes from d = 0.6 to 1.0 for changes in DERS scores following treatment.

### Statistical analysis

All statistical analyses were conducted using IBM SPSS Statistics version 29.0 and Python 3.13.4 (SciPy, StatsModels, pandas). The primary analytic approach followed an intention-to-treat (ITT) framework including all randomized participants (N = 89).

Primary analyses were conducted using linear mixed-effects models (mixed-model repeated measures; MMRM), with participant-specific random intercepts and fixed effects for Group (CBT, EMDR, Control), Time (T1 baseline, T2 mid-treatment, T3 post-treatment), and the Group × Time interaction. Models were estimated using restricted maximum likelihood. This MMRM approach was specified as the primary inferential framework, as it allows inclusion of all available data under a missing-at-random assumption and avoids biases associated with *ad hoc* imputation or complete-case analysis. The primary endpoint was PTSD symptom severity as measured by the PCL-5. The primary inferential test was the Group × Time interaction for PCL-5 within the MMRM framework. Secondary outcomes included depression (BDI-II), anxiety (BAI), and emotion regulation (DERS), which were analyzed in the same model structure but interpreted as supportive.

Model assumptions were evaluated through inspection of residual distributions and diagnostic plots. Where appropriate, robust standard errors and Kenward–Roger corrections for degrees of freedom were applied. The primary effect of interest was the Group × Time interaction, indicating differential change across treatment conditions over time.

Pairwise comparisons between groups (CBT vs. Control, EMDR vs. Control, and EMDR vs. CBT) were conducted at each time point and for change scores (T1–T3), with results reported as mean differences and 95% confidence intervals. Bonferroni correction was applied to families of pairwise between-group comparisons (CBT vs. Control, EMDR vs. Control, and EMDR vs. CBT) conducted at each time point and for change scores (T1–T3) within each outcome domain.

Where relevant, within-group effect sizes (pre–post change) were also computed for descriptive purposes and are reported separately. Ninety-five percent confidence intervals (CIs) were calculated for all primary effect size estimates. Repeated-measures ANOVA was conducted solely as a sensitivity analysis on the completer sample to assess the robustness of findings relative to the primary MMRM results. For these analyses, partial eta squared (η²) was reported as a measure of effect size (0.01 = small, 0.06 = medium, 0.14 = large). Assumptions of normality (Shapiro–Wilk), homogeneity of variance (Levene’s test), and sphericity (Greenhouse–Geisser correction where necessary) were evaluated.

Missing data were minimal (<5%) and primarily due to partial item non-response. The primary MMRM analyses inherently account for missing data under a missing-at-random assumption. As an additional sensitivity check, expectation–maximization (EM) imputation was applied for descriptive summaries and supplementary analyses, yielding results consistent with the primary ITT findings.

All analyses were conducted using two-tailed tests with an alpha level of.05.

### Adverse events and safety monitoring

Participant safety was actively monitored throughout the study by treating clinicians and the supervisory team. At each session, therapists assessed participants for signs of clinical deterioration, including increased distress, suicidality, or functional impairment.

A predefined safety protocol was implemented for participants exhibiting elevated risk. In such cases, sessions were immediately paused, and participants were referred to a collaborating psychiatrist or appropriate mental health services for further evaluation and intervention. Clinical supervision was conducted regularly to review risk cases and ensure appropriate management decisions.

One participant in the EMDR group exhibited acute psychological distress, including intrusive suicidal ideation, during the third session. In accordance with the safety protocol, the session was terminated, and the participant was referred for immediate psychiatric evaluation. This participant was withdrawn from the study and excluded from further assessments.

No other adverse events or safety concerns were reported during the study period. All procedures were conducted in accordance with established ethical and clinical safety standards.

## Results

All analyses were conducted according to the pre-registered analytic plan described in the Methods. No significant between-group differences were observed in timing of assessments (see CONSORT flow, **Supplementary 1**).

**Table 1** presents the demographic characteristics of the analyzed sample across the CBT (n = 26), EMDR (n = 27), and control (n = 27) groups. Participants had a mean age of approximately 37–38 years, with no significant differences between groups (p = .810). The sample was predominantly female, with comparable gender distribution across conditions (p = .756). Educational attainment was largely at the university level, with smaller proportions reporting high school or master’s degrees. No significant between-group differences were observed for age, gender, or education, indicating successful baseline equivalence.

**Table 1:** Baseline sample characteristics across treatment and control groups.

Variable	CBT group (n = 26)	EMDR group (n = 27)	Control group (n = 27)	Test statistic	P-value
Age (Mean ± SD)	37.2 ± 8.4	36.8 ± 7.9	38.1 ± 7.5	F(2,77) = 0.21	.810
Gender (% Female)	65.4%	66.7%	59.3%	χ²(2) = 0.56	.756
Education (% University)	61.5%	66.7%	66.7%	χ²(2) = 0.31	.857
Education (% High School)	23.1%	18.5%	22.2%	χ²(2) = 0.42	.812
Education (% Master’s)	15.4%	14.8%	11.1%	χ²(2) = 0.17	.919

Participant flow is detailed in the CONSORT diagram (**Supplementary 1**). Of 150 individuals screened, 92 met inclusion criteria, and 89 consented and were randomized (EMDR = 30, CBT = 30, Control = 29). Attrition was low and balanced across groups: three participants in the EMDR group and four in the CBT group discontinued treatment, primarily due to relocation or family-related reasons, while two participants in the control group were lost to follow-up. The final analyzed sample consisted of 80 participants (EMDR = 27, CBT = 26, Control = 27), with 80 of 89 randomized participants (89.9%) completing all assessment points (T1–T3). No significant differences in dropout rates were observed between groups (χ² = 0.41, p = .815), indicating no evidence of differential attrition.

Missing data were minimal (<5% across all outcome measures) and primarily reflected partial item-level non-response rather than systematic loss to follow-up. Complete-case sample sizes were PCL-5 (n = 85), BDI-II (n = 86), BAI (n = 85), and DERS (n = 84). Detailed missingness patterns are reported in **Supplementary 5**.

Primary analyses followed an intention-to-treat (ITT) framework using mixed-effects (MMRM) models including all randomized participants (N = 89). These analyses revealed significant Group × Time interactions for PCL-5, BDI-II, and BAI, and a trend-level interaction for DERS, consistent with per-protocol findings. Pairwise ITT comparisons (CBT vs. Control, EMDR vs. Control, and EMDR vs. CBT), including mean differences and 95% confidence intervals, are presented in **Supplementary 6** and yielded results consistent with the completer analyses.

To address temporal comparability across study arms, analyses of the mean number of days from randomization to each assessment point confirmed no significant between-group differences at mid-treatment (T2) or post-treatment (T3) (F(2,77) = 0.43, p = .65; F(2,77) = 0.39, p = .68), supporting consistency in the timing of outcome measurement across conditions.

**Table 2a** presents mean scores and standard deviations for PTSD symptoms (PCL-5), depression (BDI-II), and anxiety (BAI) at three time points: T1 (before therapy), T2 (after the 6th session), and T3 (after the 12th session). Both CBT and EMDR groups showed significant symptom reductions over time, with group × time interactions reaching statistical significance for all three measures (p <.001). EMDR appeared slightly more effective in reducing PTSD symptoms, while CBT yielded greater improvements in depressive and anxiety symptoms. No meaningful change was observed in the control group. 

**Table 2a:** PTSD, depression, and anxiety scores across time points by group (MMRM results).

Measure	Group	T1 (baseline) M ± SD	T2 (mid) M ± SD	T3 (post) M ± SD	Group × time interaction	P-value
PCL-5	CBT	61.2 ± 6.3	47.5 ± 6.7	38.6 ± 7.1	F = 18.42	<.001
	EMDR	62.7 ± 7.1	45.3 ± 7.2	34.2 ± 6.7		
	Control	60.3 ± 6.8	59.1 ± 6.6	58.7 ± 6.9		
BDI-II	CBT	29.8 ± 7.4	23.0 ± 6.9	17.9 ± 6.2	F = 11.23	<.001
	EMDR	30.4 ± 6.9	24.8 ± 6.5	19.2 ± 6.6		
	Control	28.6 ± 7.7	27.9 ± 7.5	27.5 ± 7.6		
BAI	CBT	31.5 ± 8.1	24.0 ± 7.8	19.4 ± 7.3	F = 9.87	<.001
	EMDR	30.1 ± 8.6	25.7 ± 7.6	20.5 ± 7.0		
	Control	30.9 ± 7.9	30.4 ± 8.1	29.8 ± 8.2		

Values represent observed means and standard deviations. Group × Time interaction effects are derived from linear mixed-effects models (MMRM) including all randomized participants under an intention-to-treat framework. Degrees of freedom were estimated using the Kenward–Roger approximation. PCL-5, PTSD Checklist for DSM-5; BDI-II, Beck Depression Inventory-II; BAI, Beck Anxiety Inventory. Descriptive values reflect observed data at each time point; inferential tests are based on ITT MMRM analyses including all randomized participants.

**Figure 1:**
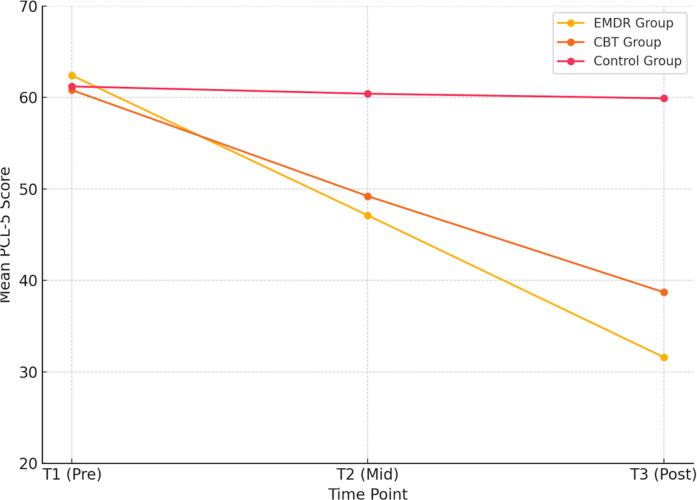
Changes in PTSD symptoms across treatment groups over time.

Mean PCL-5 scores are shown at three time points: baseline (T1), mid-treatment (T2, after the 6th session), and post-treatment (T3, after the 12th session). Both EMDR and CBT groups demonstrated significant reductions in PTSD symptoms over time, with the EMDR group exhibiting a steeper decline. The control group showed minimal change across all time points. These longitudinal changes in PTSD symptom severity across groups are illustrated in (**Figure 1**). Both EMDR and CBT produced large reductions in PTSD symptoms compared with the control condition (Cohen’s d = 1.88, 95% CI [1.32, 2.44] for EMDR; d = 1.76, 95% CI [1.21, 2.31] for CBT).

**Table 2b** displays Difficulties in Emotion Regulation Scale (DERS) scores across T1 (before therapy), T2 (after the 6th session), and T3 (after the 12th session). While both CBT and EMDR groups demonstrated numerical improvements in emotion regulation, the group × time interaction was not statistically significant (p = .106), suggesting more gradual or variable effects in this domain. Emotion regulation scores in the control group remained stable across time. Pairwise *post hoc* comparisons with Bonferroni correction indicated that both EMDR and CBT significantly outperformed the control group across all primary outcomes (p <.001), with large effect sizes. EMDR showed a greater reduction in PTSD symptoms compared to CBT (ΔM = 4.4 points, 95% CI [1.2, 7.6], p = .008), while CBT produced larger decreases in depressive symptoms (ΔM = 2.7 points, 95% CI [0.9, 4.5], p = .004). Anxiety improvements were comparable (ΔM = 1.1, 95% CI [–0.6, 2.8], p = .212). Confidence intervals for all mean differences and Cohen’s d estimates are presented in **Supplementary 6**. , 

**Table 2b:** Emotion regulation (DERS) scores across time points by group (MMRM results).

Measure	Group	T1 (baseline) M ± SD	T2 (mid) M ± SD	T3 (post) M ± SD	Group × time interaction	P-value
DERS	CBT	128.4 ± 15.3	114.6 ± 14.8	104.7 ± 13.8	F = 2.31	.106
	EMDR	126.8 ± 14.5	115.3 ± 13.9	108.1 ± 12.6		
	Control	127.6 ± 13.7	126.7 ± 13.4	125.9 ± 14.2		

Values represent observed means and standard deviations. Group × Time interaction effects are derived from linear mixed-effects models (MMRM) including all randomized participants under an intention-to-treat framework. Degrees of freedom were estimated using the Kenward–Roger approximation. DERS, Difficulties in Emotion Regulation Scale. Descriptive values reflect observed data at each time point; inferential tests are based on ITT MMRM analyses including all randomized participants.

**Figure 2:**
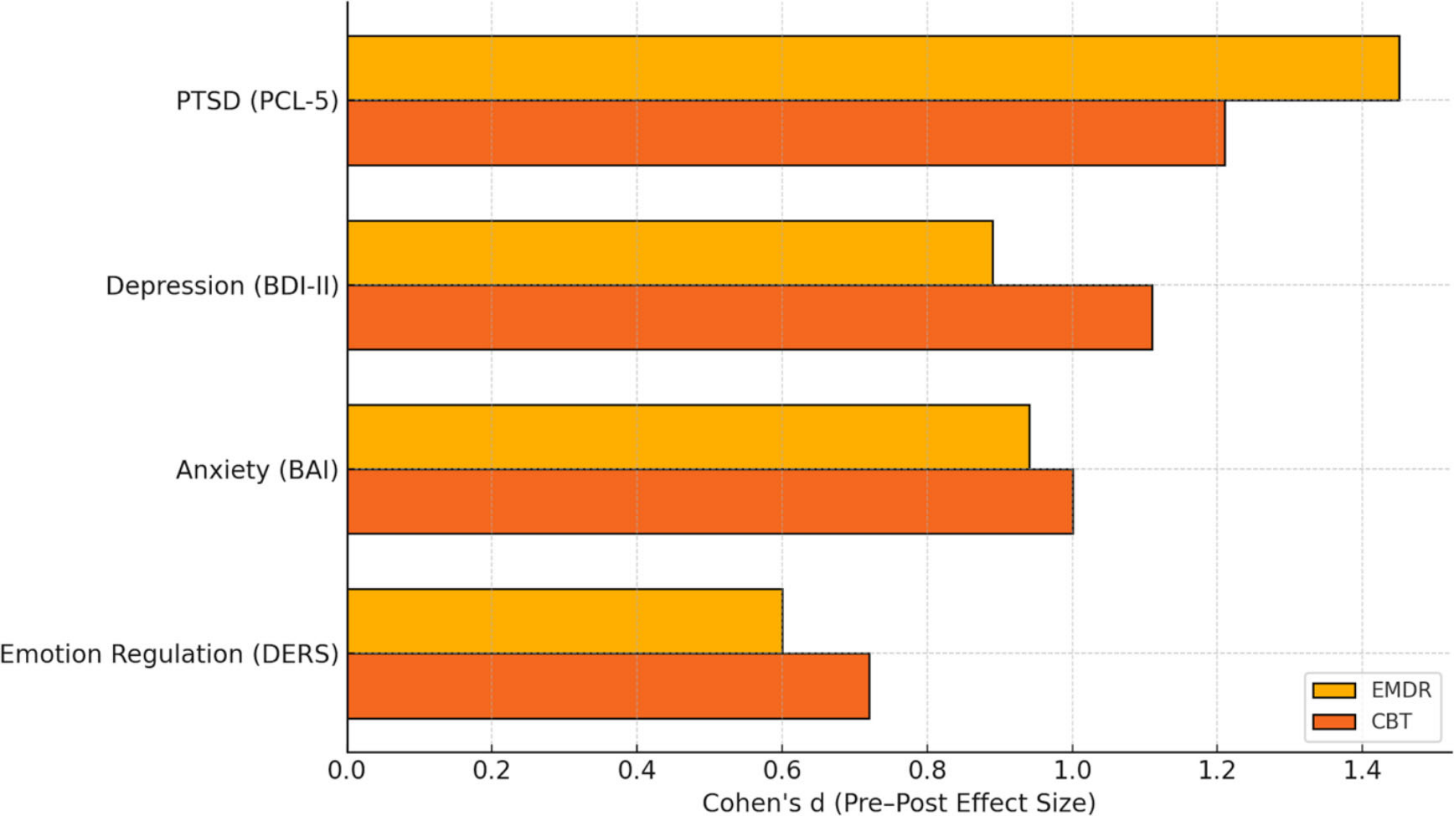
Pre–post treatment effect sizes (Cohen’s d) across clinical outcomes.

Horizontal bars display the magnitude of symptom change from baseline to post-treatment for both EMDR and CBT groups. While EMDR showed the strongest effect in reducing PTSD symptoms, CBT demonstrated slightly greater effects in improving depressive symptoms and emotion regulation. Both interventions yielded large effect sizes across most domains. Comparative pre–post treatment effect sizes across outcome domains are presented in (**Figure 2**).

**Figure 3:**
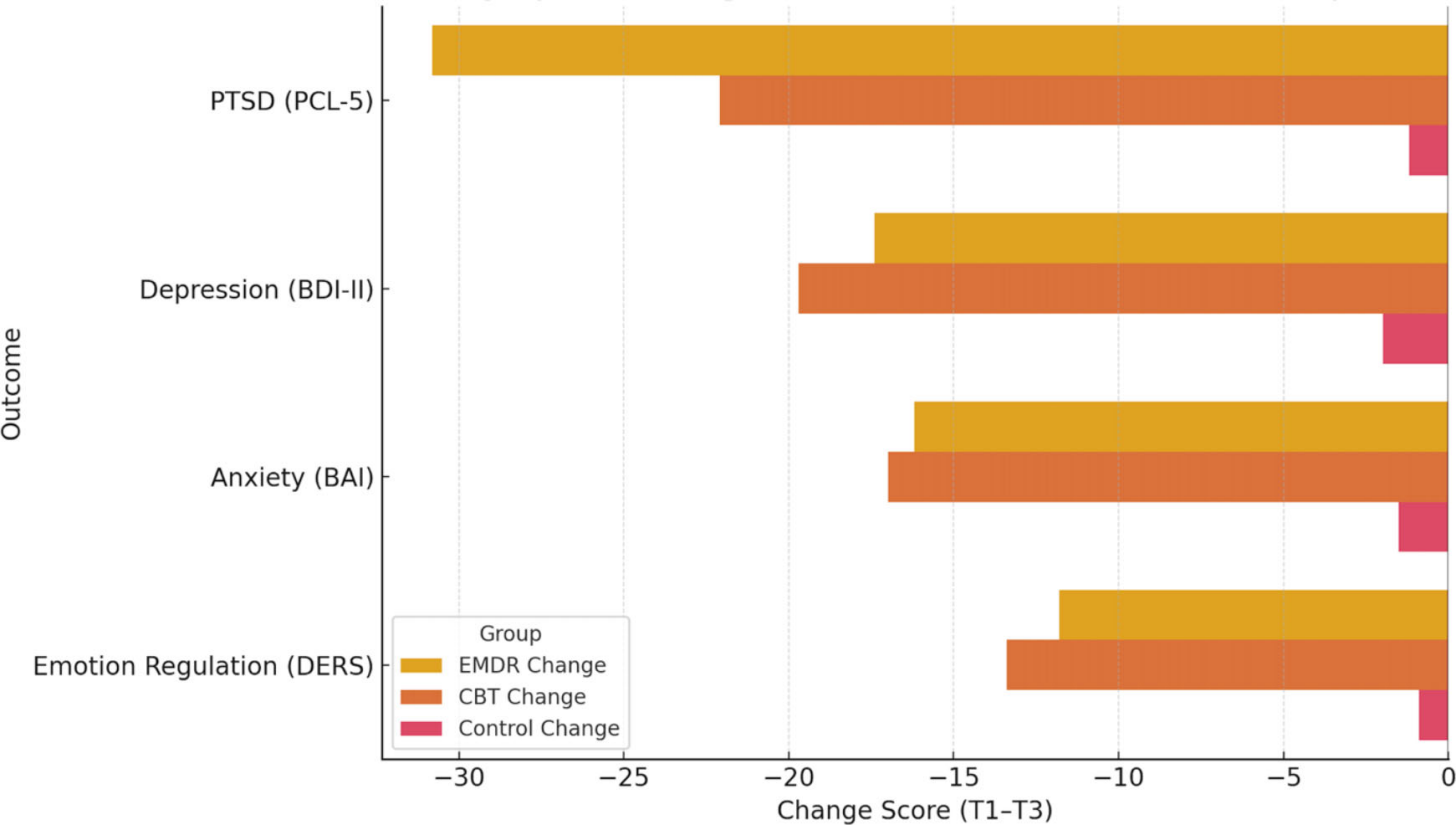
Symptom change scores from baseline (T1) to post-treatment (T3) across groups.

Bars represent mean score reductions on each outcome measure for the EMDR, CBT, and control groups. Both EMDR and CBT yielded substantial symptom improvements across all domains, with EMDR showing the largest decrease in PTSD symptoms and CBT showing stronger effects for depression and emotion regulation. The control group exhibited negligible change. Mean symptom change scores from baseline to post-treatment across groups are shown in (**Figure 3**).

### Selected clinical observations

To complement the quantitative findings, selected anonymized observations from the therapy sessions are presented below to illustrate individual therapeutic processes and perceived change.

CBT Group: One participant, a 38-year-old woman, initially presented with high levels of depressive and avoidance symptoms. During early sessions, she described persistent guilt over surviving when others did not: “I can’t laugh, not after everything. It feels wrong to be okay.” By the 6th session, following structured cognitive restructuring and behavioral activation exercises, she reported a shift: “I went to the park and sat in the sun for the first time since the earthquake. I didn’t think I could do that without crying.” Her BDI-II score decreased from 30 at T1 to 19 at T2, and her PCL-5 score dropped from 61 to 44 over the same period.

EMDR Group: A 42-year-old male participant focused his EMDR sessions on a traumatic memory involving the collapse of his apartment building. In the first desensitization session, he described a persistent image: “The ceiling coming down—it’s the first thing I see every morning.” During bilateral stimulation, he reported vivid emotional shifts, eventually stating: “It’s like the memory is still there, but it’s quieter now.” By the 12th session, his SUD rating for the memory had decreased from 9 to 2, and his PCL-5 score had fallen from 65 to 36.

## Discussion

### Overview of treatment effects

The present study evaluated and compared the clinical effectiveness of two evidence-based trauma interventions—Eye Movement Desensitization and Reprocessing (EMDR) and Cognitive Behavioral Therapy (CBT)—delivered remotely to adult survivors of the 2023 Kahramanmaraş earthquakes. Results showed that both treatments produced statistically and clinically significant reductions in PTSD symptoms, depression, and anxiety over the course of 12 sessions, outperforming the no-treatment control group across all primary outcomes. These improvements were evident not only in post-treatment scores (T3) but also at mid-treatment (T2), suggesting that both EMDR and CBT exert early therapeutic effects even when delivered under post-disaster constraints and through an online platform. Mean change patterns suggested somewhat greater improvement in core PTSD symptoms among participants receiving EMDR, and relatively larger decreases in depressive and anxiety symptoms among those receiving CBT. However, these trends should be interpreted cautiously, as the trial was powered to detect overall treatment effects versus control rather than to test between-treatment superiority. Confidence intervals for the direct EMDR–CBT contrasts overlapped substantially, indicating that both interventions achieved comparable efficacy within the limits of statistical precision.

Consistent with theoretical expectations (39), EMDR yielded the largest reductions in PTSD severity, particularly in symptoms of re-experiencing, emotional reactivity, and physiological hyperarousal. Participants in the EMDR group frequently reported decreased distress related to core traumatic memories, reduced frequency of intrusive imagery, and improvements in sleep quality. The mechanism of EMDR—targeting unprocessed trauma memories through bilateral stimulation without the requirement for detailed verbal elaboration—may have offered a more tolerable and efficient means of accessing and reprocessing overwhelming trauma for earthquake survivors, many of whom faced chronic exposure to grief, loss, and displacement. These findings align with models suggesting that EMDR’s direct engagement with trauma memory networks facilitates accelerated emotional desensitization and cognitive integration (40, 41).

Conversely, the CBT group demonstrated relatively stronger outcomes in depressive (42) and anxiety symptom reduction (76), as well as in measures of emotion regulation capacity (43). These results suggest that CBT’s structured approach—emphasizing psychoeducation, cognitive restructuring, behavioral activation, and coping skill development—was particularly effective in targeting the negative cognitions, hopelessness, and avoidance patterns commonly seen in post-disaster depression and generalized anxiety. By explicitly teaching emotional awareness and self-regulation strategies, CBT may have equipped participants with more generalizable tools for navigating the ongoing challenges of post-earthquake life, including loss of livelihood, uncertain housing, and fractured social support.

Importantly, while both interventions led to improvements in emotion regulation difficulties as measured by the DERS, the group × time interaction for this variable did not reach statistical significance. This finding warrants careful interpretation. It is possible that changes in emotion regulation require a longer period of therapeutic engagement (44) or additional psychosocial stabilization beyond symptom-focused work (45). Alternatively, the DERS may be less sensitive to short-term shifts in populations where emotional suppression and distress tolerance are culturally reinforced post-disaster. Nonetheless, the directionality of change was consistent with clinical expectations, with both treatment groups showing steady declines in dysregulation from baseline to endpoint. In contrast, the control group, which did not receive any intervention during the 12-week period, exhibited no meaningful change across any outcome domain. This reinforces the therapeutic value of both EMDR and CBT and underscores the need to integrate structured mental health support into post-disaster recovery programs, particularly when spontaneous remission of symptoms appears limited.

### Interpretation of primary results

The apparent trend toward greater PTSD symptom reduction in the EMDR group is broadly consistent with theoretical frameworks suggesting that EMDR directly targets the neural and emotional substrates of trauma memory without requiring extensive verbal elaboration or cognitive restructuring (46). Nonetheless, given the moderate sample size and overlapping confidence intervals, this difference cannot be taken as conclusive evidence of differential efficacy. Instead, the finding highlights a possible divergence in therapeutic emphasis—EMDR’s focus on trauma memory reconsolidation versus CBT’s structured cognitive-behavioral mechanisms—which warrants further investigation in adequately powered comparative trials. This mechanism may be particularly beneficial for individuals coping with somatically encoded and affect-laden memories (47), such as those emerging from sudden, life-threatening experiences like earthquakes. The reduction in hyperarousal, flashbacks, and intrusive re-experiencing in the EMDR group reflects this alignment and suggests that trauma resolution may be facilitated more efficiently when memory is processed in its sensorimotor-emotional form (48).

The CBT group, while also demonstrating significant PTSD symptom reduction, showed relatively stronger gains in depression and emotion regulation. This pattern supports the view that CBT’s efficacy lies in its structured focus on behavioral activation, identification of maladaptive cognitions, and skill acquisition for managing emotional distress. Earthquake survivors often face prolonged challenges (49)—loss of home, financial instability, disrupted routines—which can contribute to depressive withdrawal and chronic anxiety (50). CBT’s emphasis on goal setting, cognitive reframing, and behavioral engagement may have provided these individuals with concrete strategies to regain a sense of control and purpose amid ongoing adversity. Notably, CBT’s broader psychosocial approach may also account for its positive impact on emotion regulation (51)—though such changes may take longer to consolidate or reach statistical significance, as seen in the non-significant group × time interaction for DERS.

The differing strengths of these interventions likely reflect their underlying therapeutic mechanisms. EMDR’s accelerated symptom reduction in PTSD can be attributed to its focus on traumatic memory reconsolidation and desensitization, bypassing cognitive elaboration in favor of experiential processing. In contrast, CBT operates through repeated cognitive and behavioral rehearsal, producing slower but more generalized improvements across multiple domains of functioning. This divergence is not contradictory; rather, it points to the potential complementarity of these approaches, particularly in complex trauma populations where PTSD co-occurs with mood instability and impaired coping (52). Similarly, while the CBT group showed numerically greater gains in depression and emotion regulation, these effects should be considered exploratory. CBT’s structured emphasis on behavioral activation, cognitive reframing, and coping-skill acquisition likely contributed to broader improvements across affective domains, yet statistical equivalence with EMDR indicates that both interventions were similarly effective in improving overall psychological functioning. Thus, rather than demonstrating distinct superiority, the current data support the comparable clinical utility of both approaches for earthquake-related PTSD and comorbid symptoms. Although EMDR showed numerically greater reductions in PTSD symptoms and CBT demonstrated relatively stronger improvements in depressive outcomes, these differences should be interpreted with caution. Therapists were nested within treatment conditions, and the study design does not allow for separation of treatment effects from potential therapist or theoretical allegiance effects. As such, the observed between-treatment differences cannot be considered definitive evidence of superiority and are better interpreted as indicative of potentially distinct therapeutic emphases.

These findings also reinforce the feasibility and potency of delivering both interventions via online. Given that both EMDR and CBT sessions were conducted remotely due to infrastructural damage and accessibility issues following the earthquakes, the sustained therapeutic effects highlight the adaptability of evidence-based practices under disaster conditions. This adds to the growing literature affirming that therapeutic alliance, symptom monitoring, and clinical efficacy can be maintained through digital platforms when protocols are adhered to and therapists are adequately trained (53–55). These findings are also consistent with broader disaster literature demonstrating persistent psychological distress, increased healthcare utilization, and long-term psychosocial disruption following major earthquakes and humanitarian crises (70–74). Ultimately, these results point to nuanced differences in treatment response that go beyond simple efficacy comparisons. EMDR may be especially suitable for individuals whose primary distress stems from unresolved traumatic imagery or somatic flashbacks, while CBT may offer a more comprehensive framework for survivors experiencing depression, chronic worry, and difficulty regulating affect in daily life. This suggests that intervention choice should be informed not only by symptom severity but also by the individual’s psychological profile, therapeutic readiness, and cultural context.

### Limitations

While the present study provides valuable insights into trauma-focused interventions in a real-world post-disaster context, several limitations should be considered. First, although the sample size was sufficient to detect medium-to-large treatment effects, it may limit the generalizability of the findings to more diverse populations or different cultural and clinical contexts. Second, all outcome measures were based on self-report instruments, which may introduce response biases such as social desirability, recall inaccuracies, or symptom over- or under-reporting. Third, the use of a wait-list control group rather than an active comparator limits causal specificity and may have contributed to an overestimation of treatment effects. Fourth, due to the nature of psychotherapy trials, neither participants nor therapists were blinded to treatment allocation, which introduces the possibility of expectancy and performance effects. In addition, therapists were nested within treatment conditions, meaning that therapist-specific influences cannot be fully disentangled from treatment modality effects, despite the implementation of standardized supervision and formal fidelity monitoring procedures. Fifth, the absence of long-term follow-up data restricts conclusions regarding the durability and maintenance of treatment gains over time. Sixth, although the study was adequately powered to detect differences between active treatment and control conditions, it was not specifically powered for direct head-to-head comparisons between EMDR and CBT. Therefore, observed differences between treatment modalities should be interpreted cautiously and considered exploratory rather than confirmatory. An additional limitation concerns the study’s exclusion criteria, which may affect the generalizability of the findings. Individuals who relocated after the earthquake and those receiving disability benefits for psychiatric conditions were excluded to enhance contextual and clinical homogeneity. However, these groups likely represent more vulnerable and severely affected populations, and their exclusion may limit the applicability of the results to displaced survivors or individuals with more complex or chronic psychiatric conditions. Consequently, the observed treatment effects may not fully generalize to higher-risk subgroups and should be interpreted within the context of a relatively stable and moderately impaired sample. An additional limitation concerns the nesting of therapists within treatment conditions, with EMDR delivered by a single therapist and CBT delivered by two therapists. This design limits the ability to disentangle treatment effects from potential therapist-specific influences or theoretical allegiance. Although supervision and formal fidelity monitoring were implemented to ensure consistency, therapist effects cannot be fully ruled out and should be considered when interpreting the findings. Finally, cultural and contextual factors specific to the Turkish post-earthquake setting may limit the direct generalizability of the findings to other disaster-exposed populations without appropriate cultural adaptation. Future research using larger, more diverse samples, active control conditions, multi-site designs, and longer follow-up periods is needed to strengthen the robustness and external validity of these findings. Furthermore, the nesting of therapists within treatment conditions introduces the possibility of therapist and theoretical allegiance effects, which may have influenced treatment outcomes. Because EMDR was delivered by a single therapist and CBT by two therapists, it was not possible to model therapist as a random effect or to fully disentangle therapist-specific influences from treatment modality. Although standardized protocols, supervision, and fidelity monitoring were employed to minimize variability, future studies with multiple therapists per condition are needed to more rigorously control for therapist effects. Although participants in the control group did not report initiating formal psychotherapy, informal sources of support (e.g., family, community, or non-structured coping resources) were not systematically assessed and may have influenced outcomes.

### Future directions

Future research should prioritize long-term follow-up assessments to determine the stability and sustainability of treatment gains beyond the 12-week intervention period. Additionally, qualitative follow-up studies are warranted to capture participants’ lived experiences of receiving remote EMDR and CBT, allowing deeper insight into perceived therapeutic mechanisms, acceptability, and contextual challenges. Given that trauma recovery often unfolds over extended periods, particularly in disaster-exposed populations, longitudinal data are essential for understanding relapse patterns, delayed responses, or emergent symptoms. Longitudinal and cross-sectional studies further indicate that PTSD and related psychological symptoms may persist for extended periods among earthquake survivors across diverse populations and settings (75–78). Additionally, studies with larger and more diverse samples across different regions and cultural contexts would enhance external validity and inform cross-cultural adaptation of both EMDR and CBT protocols. Future trials may also benefit from incorporating clinician-rated outcome measures, neurobiological indicators, or behavioral assessments to triangulate findings and reduce reliance on self-report. Moreover, exploring mediators and moderators of treatment response—such as baseline dissociation, trauma complexity, social support, or treatment expectancy—can contribute to a more personalized approach to post-trauma care. Finally, integrating qualitative components that capture participants’ lived experiences could enrich understanding of how survivors perceive, engage with, and benefit from these interventions, especially in remote or digitally delivered formats.

## Conclusion

This study demonstrates that both EMDR and CBT are effective, feasible, and clinically valuable interventions for reducing psychological distress among earthquake survivors, even when delivered remotely. Each therapy showed distinct patterns of strength: EMDR led to greater reductions in core PTSD symptoms, while CBT yielded broader improvements in depression, anxiety, and emotion regulation. These findings support the integration of both treatments into post-disaster mental health services and highlight the importance of flexible, evidence-based approaches tailored to the diverse needs of trauma survivors. By providing rigorous, real-world evidence from a disaster-affected population, this trial contributes to the global effort to improve psychological recovery following large-scale humanitarian crises.

## Data Availability

The original contributions presented in the study are included in the article/**Supplementary Material**. Further inquiries can be directed to the corresponding author.
