# Supplementary material for: Remote EMDR versus CBT for PTSD after the Kahramanmaraş earthquakes: a randomized trial
Source: Front Psychiatry. 2026 May 22;17:1779057. doi: 10.3389/fpsyt.2026.1779057 (PMC13236641; doi:10.3389/fpsyt.2026.1779057)
Supplement: Supplementary file 1 [file Table1.docx]

**CONSORT FLOW DIAGRAM**

Analyses control group (n=27)
Repeated-measures ANOVA, independent t-tests, Cohen’s d, and expectation-maximization algorithm for missing data.

Analyses CBT study group (n=26)
Analyses EMDR study group (n=27)
Repeated-measures ANOVA, independent t-tests, Cohen’s d, and expectation-maximization algorithm for missing data.

Excluded =(61)
* Low PTSD
* Other Mental Conditions
* Drop outs

**Assessment**

**Withdrawals**

**Analyses**

**Enrollment**

Initial assessment (T1-Pre), Subsequent Assessments (T2-Mid, T3-Post): PTSD Checklist for DSM, Beck Depression Inventory-II, Beck Anxiety Inventory, Dificulties in Emotion Regulation Scale

Initial assessment (T1-Pre), Subsequent Assessments (T2-Mid, T3-Post): PTSD Checklist for DSM, Beck Depression Inventory-II, Beck Anxiety Inventory, Dificulties in Emotion Regulation Scale

Withdrawals from control group (n=2)
Remained n=27

Withdrawals from CBT study group (n=4) Remained (n=26)
Withdrawals from EMDR study group (n=3) Remained (n=27)

**Allocation**

Study groups
CBT intervention (n=30)
EMDR intervention (n=30)

Control group
No intervention (n=29)

Randomized=89

Assessed for eligibility (n=150)
