# Supplementary material for: Remote EMDR versus CBT for PTSD after the Kahramanmaraş earthquakes: a randomized trial
Source: Front Psychiatry. 2026 May 22;17:1779057. doi: 10.3389/fpsyt.2026.1779057 (PMC13236641; doi:10.3389/fpsyt.2026.1779057)
Supplement: Supplementary file 2 [file Table2.docx]

Supplementary Table Fidelity Monitoring Checklist Scores by Independent Quality Assurance Committee

| Participant ID | Therapy Type | Session Reviewed | Adherence to Protocol (0–2) | Therapist Competence (0–2) | Session Integrity (0–2) | Comments by Committee |
| --- | --- | --- | --- | --- | --- | --- |
| CBT-03 | CBT | Session 4 | 2 | 2 | 2 | Fully adherent; excellent pacing. |
| CBT-11 | CBT | Session 7 | 2 | 1 | 2 | Slight deviation in cognitive restructuring section. |
| CBT-19 | CBT | Session 10 | 2 | 2 | 2 | Strong exposure component; good rapport. |
| EMDR-05 | EMDR | Session 3 | 2 | 2 | 2 | Correct BLS timing and phase transitions. |
| EMDR-14 | EMDR | Session 6 | 2 | 2 | 1 | Minor delay in closure; otherwise complete. |
| EMDR-22 | EMDR | Session 9 | 1 | 1 | 2 | Skipped somatic check; feedback provided. |

Scoring System:
0 = Not demonstrated; 1 = Partially demonstrated; 2 = Fully demonstrated
