# Supplementary material for: Remote EMDR versus CBT for PTSD after the Kahramanmaraş earthquakes: a randomized trial
Source: Front Psychiatry. 2026 May 22;17:1779057. doi: 10.3389/fpsyt.2026.1779057 (PMC13236641; doi:10.3389/fpsyt.2026.1779057)
Supplement: Supplementary file 3 [file Table3.docx]

Supplementary Appendix A. EMDR Intervention Structure (12 Sessions)

| Session | Focus | Description |
| --- | --- | --- |
| Session 1 | Phase 1: History Taking & Case Conceptualization | Gathering trauma history, identifying target memories, establishing the client’s readiness, and formulating a case plan according to the standard EMDR framework. |
| Session 2 | Phase 2: Preparation | Introducing the EMDR model, teaching stabilization techniques (e.g., Safe Place, Butterfly Hug), and explaining bilateral stimulation (BLS) procedures adapted for remote delivery. |
| Session 3 | Phase 2 (continued): Stabilization and Resource Installation | Practicing self-regulation and grounding techniques to ensure emotional safety during trauma processing. Therapist monitors window of tolerance. |
| Session 4 | Phase 3: Assessment | Selecting the first target memory, identifying the associated image, negative and positive cognitions, validity of cognition (VOC), and subjective units of distress (SUD). |
| Session 5 | Phase 4: Desensitization | Initiating BLS (e.g., eye movements via screen, self-tapping) while the client holds the trauma target in mind. The process continues until SUD levels are reduced. |
| Session 6 | Phase 4 (continued): Desensitization | Continuing BLS for the same or a new target memory. Therapist supports spontaneous associations and reprocessing. SUD monitored after each BLS set. |
| Session 7 | Phase 5: Installation of Positive Cognition | Strengthening the preferred positive belief (e.g., “I survived,” “I am safe now”) using BLS until VOC is maximized and consistent. |
| Session 8 | Phase 6: Body Scan | Client focuses on residual somatic sensations related to the processed memory. BLS is used to clear any remaining tension or disturbance. |
| Session 9 | Phase 4–6: Additional Target Processing | Repeating the assessment, desensitization, installation, and body scan for a second or third trauma-related memory. |
| Session 10 | Phase 7: Closure | Using self-soothing strategies to close each session. Review of progress, grounding exercises, and re-orienting to the present moment. |
| Session 11 | Phase 8: Reevaluation | Reviewing target memories processed, checking for residual distress, and assessing whether new targets have emerged. |
| Session 12 | Reevaluation and Termination | Final review of treatment progress, discussion of remaining themes, reinforcement of gains, and planning for post-treatment stability or referrals if needed. |
