# Supplementary material for: Remote EMDR versus CBT for PTSD after the Kahramanmaraş earthquakes: a randomized trial
Source: Front Psychiatry. 2026 May 22;17:1779057. doi: 10.3389/fpsyt.2026.1779057 (PMC13236641; doi:10.3389/fpsyt.2026.1779057)
Supplement: Supplementary file 4 [file Table4.docx]

Supplementary, CBT Intervention Structure (12 Sessions)

| Session | Focus | Description |
| --- | --- | --- |
| Session 1 | Introduction and Psychoeducation | Establishing therapeutic rapport, explaining the cognitive-behavioral model of PTSD, normalizing trauma responses, and introducing treatment rationale. |
| Session 2 | Case Formulation and Goal Setting | Developing an individualized case formulation, identifying core trauma-related beliefs, and collaboratively setting short- and long-term therapy goals. |
| Session 3 | Emotional Awareness and Coping Skills | Teaching basic emotion regulation techniques such as deep breathing, grounding, and thought labeling to manage acute distress. |
| Session 4 | Behavioral Activation | Identifying avoidance patterns and introducing pleasurable or meaningful activities to counter withdrawal, hopelessness, and anhedonia. |
| Session 5 | Cognitive Restructuring I | Identifying automatic negative thoughts related to the trauma, and challenging distorted appraisals using Socratic questioning and thought records. |
| Session 6 | Cognitive Restructuring II | Deepening work on core beliefs (e.g., “I am weak,” “The world is dangerous”) and replacing them with balanced, adaptive alternatives. |
| Session 7 | Avoidance and Safety Behaviors | Mapping and gradually confronting behavioral and cognitive avoidance (e.g., hypervigilance, isolation) through behavioral experiments. |
| Session 8 | Exposure Preparation | Preparing the client for imaginal exposure: identifying key traumatic memories, constructing trauma narratives, and explaining the exposure process. |
| Session 9 | Imaginal Exposure I | First guided imaginal exposure to the trauma memory, with the therapist facilitating detailed verbal recounting and emotional processing. |
| Session 10 | Imaginal Exposure II | Continued exposure with real-time monitoring of distress levels, followed by processing and integration of new cognitive insights. |
| Session 11 | Relapse Prevention I | Reviewing therapeutic gains, identifying remaining triggers or vulnerabilities, and reinforcing coping strategies. |
| Session 12 | Relapse Prevention II and Closure | Developing a personalized relapse prevention plan, reinforcing self-efficacy, and discussing termination and future support options. |
