# Supplementary material for: Remote EMDR versus CBT for PTSD after the Kahramanmaraş earthquakes: a randomized trial
Source: Front Psychiatry. 2026 May 22;17:1779057. doi: 10.3389/fpsyt.2026.1779057 (PMC13236641; doi:10.3389/fpsyt.2026.1779057)
Supplement: Supplementary file 5 [file Table5.docx]

**Supplementary 5. Percentage of Missing Data Across Measures and Time Points**

| **Measure** | **Baseline (T1)** | **Mid-treatment (T2)** | **Post-treatment (T3)** | **Total Missing (%)** |
| --- | --- | --- | --- | --- |
| PTSD Checklist for DSM-5 (PCL-5) | 0% | 3.4% (n = 3) | 4.5% (n = 4) | 2.6% |
| Beck Depression Inventory-II (BDI-II) | 0% | 2.2% (n = 2) | 4.5% (n = 4) | 2.2% |
| Beck Anxiety Inventory (BAI) | 0% | 3.4% (n = 3) | 3.4% (n = 3) | 2.3% |
| Difficulties in Emotion Regulation Scale (DERS) | 0% | 3.4% (n = 3) | 5.6% (n = 5) | 3.0% |

**Notes:**

1. Missing data were minimal across all measures (< 5%) and occurred primarily due to incomplete online self-report submissions or intermittent connectivity problems during remote data collection.
2. Expectation–maximisation (EM) imputation was applied under the assumption of missing at random (MAR) to generate a complete dataset for main analyses.
3. Sensitivity analyses comparing imputed and non-imputed datasets yielded equivalent significance levels and effect sizes across all primary and secondary outcomes.
4. Percentages are calculated relative to the full randomized sample (N = 89).
