# Supplementary material for: Remote EMDR versus CBT for PTSD after the Kahramanmaraş earthquakes: a randomized trial
Source: Front Psychiatry. 2026 May 22;17:1779057. doi: 10.3389/fpsyt.2026.1779057 (PMC13236641; doi:10.3389/fpsyt.2026.1779057)
Supplement: Supplementary file 6 [file Table6.docx]

**Supplementary 6. Between-Group Mean Differences, Effect Sizes, and 95 % Confidence Intervals (ITT, MMRM)**

| **Measure** | **Comparison** | **ΔM (Mean Difference)** | **95 % CI** | **Cohen’s d** |
| --- | --- | --- | --- | --- |
| PCL-5 | EMDR vs CBT | 4.4 | [1.2, 7.6] | 0.68 |
| PCL-5 | EMDR vs Control | 24.5 | [19.7, 29.3] | 2.03 |
| PCL-5 | CBT vs Control | 20.1 | [15.4, 24.8] | 1.76 |
| BDI-II | CBT vs EMDR | –2.7 | [–4.5, –0.9] | 0.63 |
| BDI-II | CBT vs Control | 9.6 | [6.8, 12.4] | 1.51 |
| BDI-II | EMDR vs Control | 6.9 | [4.1, 9.7] | 1.22 |
| BAI | CBT vs EMDR | –1.1 | [–2.8, 0.6] | 0.23 |
| BAI | CBT vs Control | 10.4 | [7.3, 13.5] | 1.34 |
| BAI | EMDR vs Control | 9.3 | [6.1, 12.5] | 1.29 |
| DERS | CBT vs EMDR | –3.4 | [–7.2, 0.4] | 0.39 |
| DERS | CBT vs Control | 21.2 | [14.5, 27.9] | 1.43 |
| DERS | EMDR vs Control | 17.8 | [11.3, 24.3] | 1.28 |

**Note.** Mean differences (ΔM) represent change scores from baseline to post-treatment (T1–T3). Positive values indicate greater symptom reduction. Analyses use **intention-to-treat (ITT)** mixed-model repeated-measures (MMRM) estimates under a missing-at-random assumption. Confidence intervals are 95 %, bias-corrected and Bonferroni-adjusted. **Per-protocol (completer-only)** results are reported separately in *Supplementary 6a* as a sensitivity analysis; conclusions are unchanged.
